# Protocol for a human placental explant model to study acute responses to pathogens

**DOI:** 10.1016/j.xpro.2025.104097

**Published:** 2025-09-17

**Authors:** Elias R. Ruiz-Morales, Regina Hoo, Ross F. Waller, Marcus C.S. Lee, David Fernandez-Antoran, Iva Kelava, Roser Vento-Tormo

**Affiliations:** 1 Wellcome Sanger Institute, Cambridge, UK; 2 Centre for Trophoblast Research, University of Cambridge, Cambridge, UK; 3 Department of Biochemistry, University of Cambridge, Cambridge, UK; 4 Wellcome Centre for Anti-Infectives Research, Division of Biological Chemistry and Drug Discovery, University of Dundee, Dundee, UK; 5 Wellcome Trust/Cancer Research UK, The Gurdon Institute, University of Cambridge, Cambridge, UK; 6 Department of Pathology, University of Cambridge, Cambridge, UK; 7 Fundacion ARAID, Zaragoza and IIS-Aragon, Zaragoza, Spain; 8 Cambridge Stem Cell Institute, University of Cambridge, Cambridge, UK

**Keywords:** Cell culture, Single Cell, Genomics, Immunology, Microbiology, Organoids

## Abstract

Here, we present a protocol for studying infections caused by pathogens associated with intrauterine complications—*Listeria monocytogenes*, *Plasmodium falciparum*, and *Toxoplasma gondii*—using an *ex vivo* placental explant model. We describe procedures for pathogen culture, preparation of placental explants using an air-liquid interface system, and infection assays. We detail procedures for tissue dissociation and CD45+ cell enrichment. This protocol allows short-term placental infection studies.

For complete details on the use and execution of this protocol, please refer to Hoo et al.^1^

## Highlights

• Guidelines for culturing *L. monocytogenes*, *P. falciparum*, and *T. gondii*
• Setup of a placental explant model supporting viability and cell preservation
• Placental explant model for acute infection with human pathogens
• Placental dissociation into single-cell suspension and immune cell enrichment

## Abstract (editor highlights)

Publisher’s note: Undertaking any experimental protocol requires adherence to local institutional guidelines for laboratory safety and ethics.

## Graphical abstract

**Figure:**
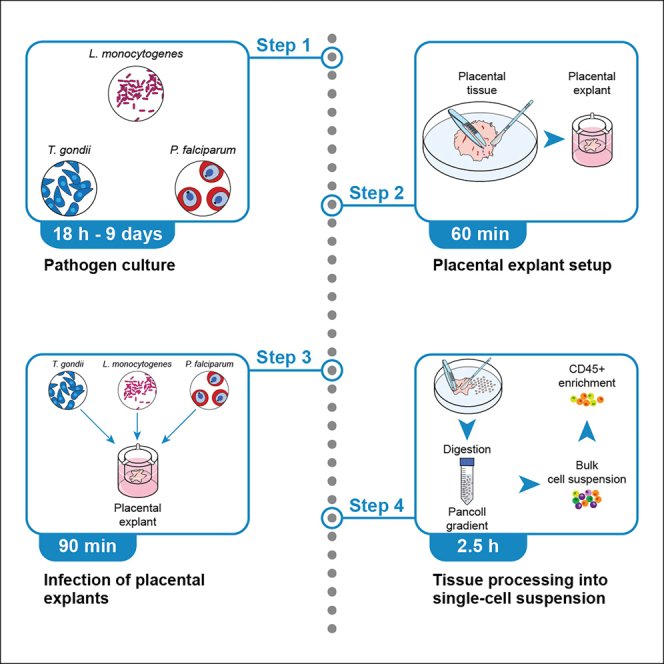

## Before you begin

This protocol describes the setup and use of an *ex vivo* placental explant model for studying placental responses to infections caused by *Listeria monocytogenes* (*L. monocytogenes*)*, Plasmodium falciparum* (*P. falciparum*), and *Toxoplasma gondii* (*T. gondii*). This method leverages an air-liquid interface culture system to maintain tissue viability and facilitate infection studies. Compared to other *ex vivo* tissue culture systems, the explant model described in this protocol enables the culture of larger placental explants that maintain key traits of trophoblasts and Hofbauer cells, and maintaining high viability of all cell types for up to 72 h. Moreover, this model has been deeply characterized at the single-cell level.^1^ The workflow presented here is designed for use with early-pregnancy human placental tissue to model acute placental responses to infections and *P. falciparum* adaptation to the placental microenvironment.^1^

The following major steps make up the protocol: (1) culture of *L. monocytogenes*, *P. falciparum*, and *T. gondii*, (2) setup of the placental explant system, (3) infection of placental explants, (4) dissociation of placental tissue into a single-cell suspension, and (5) immunomagnetic enrichment of CD45+ cells. All work with placental tissue should be performed in biosafety level 2 facilities to reduce the risk of tissue contamination and protect from unknown infections carried by the tissue. Moreover, sections involving infectious pathogens or infected tissue must be conducted according to the relevant local biosafety procedures (e.g., containment level 2 or 3 in the United Kingdom, biosafety level 2 or 3 in the United States of America).

In addition to the applications described, this protocol can be adapted for infection using other pathogens, serving as a platform to study placental and pathogen adaptations during infection. Furthermore, it can be used to study various aspects of placental biology, such as the dynamics of the placental macrophages (Hofbauer cells) and trophoblast invasion dynamics. Similarly, our air-liquid interface setup can be utilized to grow organoid models or establish explants from other tissues, enabling diverse research applications.

### Institutional permissions

All tissue samples were obtained with written informed consent from all participants in accordance with the guidelines in the Declaration of Helsinki 2000. Placental samples from elective terminations were provided by the Joint MRC/Wellcome Trust (grant #MR/R006237/1) Human Developmental Biology Resource (HDBR, http://www.hdbr.org), with appropriate maternal written consent and approval from the Fulham Research Ethics Committee (REC ref. 18/LO/0822). The HDBR is regulated by the UK Human Tissue Authority (HTA: www.hta.gov.uk) and operates in accordance with the relevant HTA Codes of Practice. Any subsequent secondary publications arising from our data generated from HDBR material should similarly acknowledge the HDBR. Ensure that your work is in compliance with all the necessary ethical requirements of your institution.

## Key resources table

**Table:** 

REAGENT or RESOURCE	SOURCE	IDENTIFIER
**Antibodies**		

CD138 (Syndecan-1) FITC antibody, anti-human, REAfinity (1:100)	Miltenyi Biotec	130-115-478; REA929
Anti-green fluorescent protein (GFP; 1:200)	Abcam	ab13970
Anti-*Listeria monocytogenes* (1:50)	Abcam	ab35132
Anti-*Toxoplasma gondii* (1:1,000)	Abcam	ab23507
CD138 (SDC1) PE antibody (1:200)	Miltenyi Biotec	130-119-840; 44F9
FITC anti-human CD45 (1:40)	BioLegend	304006; HI30
LIVE/DEAD fixable near-IR dead cell stain	Thermo Fisher Scientific	L10119
Alexa Fluor 488 anti-human CD64 antibody (1:200)	BioLegend	305010;10.1
Recombinant anti-vimentin antibody (1:350)	Abcam	ab92547, EPR3776
Anti-HLA-G antibody (MEM-G/9) PE (1:25)	Abcam	ab24384
Goat anti-rabbit IgG (H+L) cross-adsorbed secondary antibody, Alexa Fluor 488 (1:600)	Invitrogen	A11008
Donkey anti-goat IgG (H+L) cross-adsorbed secondary antibody, Alexa Fluor 647 (1:600)	Invitrogen	A21447
Donkey anti-rabbit IgG (H+L) highly cross-adsorbed secondary antibody, Alexa Fluor Plus 555 (1:600)	Invitrogen	A32794
Goat anti-mouse IgG H&L (Alexa Fluor 594) preadsorbed (1:600)	Abcam	ab150120

**Biological samples**		

Human placental tissue	Human Developmental Biology Resource United Kingdom	http://www.hdbr.org

**Chemicals, peptides, and recombinant proteins**		

Advanced DMEM/F12	Gibco	12634-010
B27, vitamin A	Life Technologies	12587010
N2	Life Technologies	17502048
Primocin	InvivoGen	ant-pm-1
N-Acetyl-L-cysteine	Sigma-Aldrich	A9165-5G
L-glutamine	Sigma-Aldrich	25030-024
Recombinant human EGF	PeproTech	AF-100-15
CHIR99021	Tocris	4423
Recombinant human R-spondin-1	R&D Systems	4645-RS-01M/CF
Recombinant human FGF-2	PeproTech	100-18B
Recombinant human HGF	PeproTech	100-39
A83-01	Tocris	2939
Prostaglandin E_2_	Sigma-Aldrich	P0409
Y-27632	Millipore	688000
FBS (fetal bovine serum)	Sigma-Aldrich	F7524
Matrigel	Corning	CLS356255
Ham’s F12 medium	PAN-Biotech	P04-14500
Penicillin-streptomycin	Gibco	15140122
DMEM, high glucose	Gibco	41965062
Amphotericin B	Gibco	15290026
Brain heart infusion broth	Millipore	53286
RPMI 1640 medium	Gibco	11875168
Albumax-II	Gibco	11021029
Hypoxanthine	Sigma-Aldrich	H9377
HEPES	Sigma-Aldrich	H4034
GlutaMAX	Gibco	35050061
Sodium chloride	Sigma-Aldrich	S5886
D-(+)-glucose (dextrose)	Sigma-Aldrich	G7021
D-sorbitol	Sigma-Aldrich	S3889
Percoll	Sigma-Aldrich	P1644
RPMI-1640 medium (powder)	Sigma-Aldrich	R6504
KnockOut serum replacement	Gibco	10828028
Trypsin-EDTA 0.25%, phenol red	Gibco	25200072
PBS pH 7.4 (1X)	Gibco	10010-015
Gentamicin	Gibco	15750060
Cell recovery solution	Corning	CLS354253
Trypsin (1:250), powder	Gibco	27250018
EDTA	Thermo Scientific	17892
Collagenase V	Sigma-Aldrich	C9263-100MG
DNase I	Roche	10104159001
Pancoll human, density: 1.077 g/mL	PAN-Biotech	P04-60500
BSA (7.5%)	Sigma	A8412-100ML
CD45 MicroBeads, human	Miltenyi Biotec	130-045-801
LS columns	Miltenyi Biotec	130-042-401
MACS BSA stock solution	Miltenyi Biotec	130-091-376

**Experimental models: Cell lines**		

Human foreskin fibroblast (HFF)	ATCC	ATCC-CRL-2429

**Experimental models: Organisms/strains**		

*Plasmodium falciparum* 2D9 NF54-eGFP	Hoo, Ruiz-Morales, Kelava, et al.^1^	N/A
*Toxoplasma gondii* RH (wild type)	Ross F. Waller	N/A
*Listeria monocytogenes* EGD-cGFP	Balestrino et al.^2^	N/A

**Other**		

Biowave (spectrophotometer)	Biochrom	CO8000
Spectrophotometer cuvettes, polystyrene	Supelco	C5677
EVOS (microscope)	Thermo Scientific	M5000
ThinCert 0.4 μm pore diameter 6-well plate inserts	Greiner Bio-One	657640
CELLSTAR 6-well cell culture multiwell plates	Greiner Bio-One	657160
Petri dish (150 mm diameter)	Sigma-Aldrich	P5981
Petri dish (69 mm diameter)	Sigma-Aldrich	P5481
Scalpel no. 20	Swann-Morton	0506
Forceps	Unomedical	84011182
25 cm^2^ rectangular canted neck cell culture flask (T-25 flask)	Corning	430639
Cell scraper	TPP	99002
27 gauge needle	BD Microlance	302200
BD sterile syringe 10 mL	BD Emerald	307736
Conical sterile tube 15 mL	Falcon	10773501
Scissors	N/A	N/A
3 μm filter	Millipore	SSWP04700
70 μm cell strainer	Falcon	352350
40 μm cell strainer	Falcon	352340
pluriStrainer Mini 20 μm (cell strainer)	pluriSelect	43-10020-60
QuadroMACS separator	Miltenyi Biotec	130-090-976
MACS MultiStand	Miltenyi Biotec	130-042-303

## Materials and equipment

### Biowave CO8000 configuration

This spectrophotometer is a cell density meter used to measure the optical density of bacteria at 600 nm (OD_600nm_). It requires 10 mm spectrophotometer cuvettes.

**Table:** Trophoblast organoid medium (TOM)

Reagent	Final concentration	Amount
Advanced DMEM/F12	N/A	8.51 mL
B27 -Vitamin A (50X)	1X	200 μL
N2 (100X)	1X	100 μL
Primocin (50 mg/mL)	100 μg/mL	20 μL
N-Acetyl-L-cysteine (500 mM)	1.25 mM	25 μL
L-glutamine (100X)	2 mM	100 μL
Recombinant human R-spondin-1 (80 μg/mL)	80 ng/mL	10 μL
Recombinant human EGF (100 μg/mL)	50 ng/mL	5 μL
Recombinant human FGF-2 (200 μg/mL)	100 ng/mL	5 μL
Recombinant human HGF (100 μg/mL)	50 ng/mL	5 μL
A83-01 (1 mM)	500 nM	5 μL
Y-27632 (10 mM)	2 μM	4 μL
CHIR99021 (15 mM)	1.5 μM	1 μL
Prostaglandin E_2_ (2.5 mM)	2.5 μM	10 μL
FBS (heat inactivated)	10%	1 mL
Total	N/A	10 mL

***Note:*** FBS must be heat inactivated by placing it in a water bath at 56°C for 30 min. Keep sterile. Store at −20°C for up to 12 months.

***Note:*** TOM^3^ is a medium designed to support trophoblast cells (the epithelial-like cells in the placenta). In our protocol, we supplement TOM with 10% FBS to support other cell lineages in the placental tissue, such as stromal and immune cells. Prepare fresh TOM every time. Store at 4°C for up to 3 days.

**Table:** TOM-Lm (*L. monocytogenes*)

Reagent	Final concentration	Amount
Advanced DMEM/F12	N/A	8.37 mL
B27 -Vitamin A (50X)	1X	200 μL
N2 (100X)	1X	100 μL
Amphotericin B	4 μg/mL	160 μL
N-Acetyl-L-cysteine (500 mM)	1.25 mM	25 μL
L-glutamine (100X)	2 mM	100 μL
Recombinant human R-spondin-1 (80 μg/mL)	80 ng/mL	10 μL
Recombinant human EGF (100 μg/mL)	50 ng/mL	5 μL
Recombinant human FGF-2 (200 μg/mL)	100 ng/mL	5 μL
Recombinant human HGF (100 μg/mL)	50 ng/mL	5 μL
A83-01 (1 mM)	500 nM	5 μL
Y-27632 (10 mM)	2 μM	4 μL
CHIR99021 (15 mM)	1.5 μM	1 μL
Prostaglandin E_2_ (2.5 mM)	2.5 μM	10 μL
FBS (heat inactivated)	10%	1 mL
Total	N/A	10 mL

***Note:*** Store at 4°C for up to 3 days.

***Note:*** FBS must be heat inactivated by placing it in a water bath at 56°C for 30 min. Keep sterile. Store at −20°C for up to 12 months.

**Table:** TOM-Lm supplemented with Gentamicin

Reagent	Final concentration	Amount
Advanced DMEM/F12	N/A	8.36 mL
B27 -Vitamin A (50X)	1X	200 μL
N2 (100X)	1X	100 μL
Amphotericin B	4 μg/mL	160 μL
N-Acetyl-L-cysteine (500 mM)	1.25 mM	25 μL
L-glutamine (100X)	2 mM	100 μL
Recombinant human R-spondin-1 (80 μg/mL)	80 ng/mL	10 μL
Recombinant human EGF (100 μg/mL)	50 ng/mL	5 μL
Recombinant human FGF-2 (200 μg/mL)	100 ng/mL	5 μL
Recombinant human HGF (100 μg/mL)	50 ng/mL	5 μL
A83-01 (1 mM)	500 nM	5 μL
Y-27632 (10 mM)	2 μM	4 μL
CHIR99021 (15 mM)	1.5 μM	1 μL
Prostaglandin E_2_ (2.5 mM)	2.5 μM	10 μL
FBS (heat inactivated)	10%	1 mL
Gentamicin	50 μg/mL	10 μL
Total	N/A	10 mL

***Note:*** Store at 4°C for up to 3 days.

***Note:*** FBS must be heat inactivated by placing it in a water bath at 56°C for 30 min. Keep sterile. Store at −20°C for up to 12 months.

**Table:** TOM-Pf (*P. falciparum*)

Reagent	Final concentration	Amount
Advanced DMEM/F12	N/A	8.03 mL
B27 -Vitamin A (50X)	1X	200 μL
N2 (100X)	1X	100 μL
N-Acetyl-L-cysteine (500 mM)	1.25 mM	25 μL
L-glutamine (100X)	2 mM	100 μL
Recombinant human R-spondin-1 (80 μg/mL)	80 ng/mL	10 μL
Recombinant human EGF (100 μg/mL)	50 ng/mL	5 μL
Recombinant human FGF-2 (200 μg/mL)	100 ng/mL	5 μL
Recombinant human HGF (100 μg/mL)	50 ng/mL	5 μL
A83-01 (1 mM)	500 nM	5 μL
Y-27632 (10 mM)	2 μM	4 μL
CHIR99021 (15 mM)	1.5 μM	1 μL
Prostaglandin E_2_ (2.5 mM)	2.5 μM	10 μL
FBS (heat inactivated)	10%	1 mL
Hematocrit	5%	0.5 mL
Total	N/A	10 mL

***Note:*** Store at 4°C for up to 3 days.

***Note:*** FBS must be heat inactivated by placing it in a water bath at 56°C for 30 min. Keep sterile. Store at −20°C for up to 12 months.

**Table:** TOM-Tg (*T. gondii*)

Reagent	Final concentration	Amount
Advanced DMEM/F12	N/A	8.325 mL
B27 -Vitamin A (50X)	1X	200 μL
N2 (100X)	1X	100 μL
Amphotericin B	4 μg/mL	160 μL
Penicillin-streptomycin (10,000 U/mL)	50 U/mL	50 μL
N-Acetyl-L-cysteine (500 mM)	1.25 mM	25 μL
L-glutamine (100X)	2 mM	100 μL
Recombinant human R-spondin-1 (80 μg/mL)	80 ng/mL	10 μL
Recombinant human EGF (100 μg/mL)	50 ng/mL	5 μL
Recombinant human FGF-2 (200 μg/mL)	100 ng/mL	5 μL
Recombinant human HGF (100 μg/mL)	50 ng/mL	5 μL
Y-27632 (10 mM)	2 μM	4 μL
CHIR99021 (15 mM)	1.5 μM	1 μL
Prostaglandin E_2_ (2.5 mM)	2.5 μM	10 μL
FBS (heat inactivated)	10%	1 mL
Total	N/A	10 mL

***Note:*** Store at 4°C for up to 3 days.

***Note:*** FBS must be heat inactivated by placing it in a water bath at 56°C for 30 min. Keep sterile. Store at −20°C for up to 12 months.

**Table:** Ham’s F12 medium penicillin-streptomycin

Reagent	Final concentration	Amount
Ham’s F12 medium	N/A	497.5 mL
Penicillin-streptomycin	50 U/mL	2.5 mL
Total	N/A	500 mL

***Note:*** Store at 4°C for up to 30 days.

**Table:** Brain heart infusion (BHI) broth

Reagent	Final concentration	Amount
Distilled water	N/A	1000 mL
Brain Heart Infusion Broth	37 g/L	37 g

***Note:*** Autoclave BHI broth at 121°C for 15 min. Store at 4°C for up to 30 days.

**Table:** D10 media

Reagent	Final concentration	Amount
DMEM, high glucose	N/A	442 mL
FBS (heat inactivated)	10%	50 mL
L-glutamine	1%	5 mL
Penicillin-streptomycin	50 U/mL	2.5 mL
Amphotericin B	0.1%	0.5 mL
Total	N/A	500 mL

***Note:*** Store at 4°C for up to 30 days.

***Note:*** FBS must be heat inactivated by placing it in a water bath at 56°C for 30 min. Keep sterile. Store at −20°C for up to 12 months.

**Table:** ED1 media

Reagent	Final concentration	Amount
DMEM, high glucose	N/A	491.5 mL
FBS (heat inactivated)	1%	5 mL
L-glutamine	0.1%	0.5 mL
Penicillin-streptomycin	50 U/mL	2.5 mL
Amphotericin B	0.1%	0.5 mL
Total	N/A	500 mL

***Note:*** Store at 4°C for up to 30 days.

***Note:*** FBS must be heat inactivated by placing it in a water bath at 56°C for 30 min. Keep sterile. Store at −20°C for up to 12 months.

**Table:** Complete RPMI (cRPMI)

Reagent	Final concentration	Amount
RPMI 1640 Medium	N/A	94 mL
Albumax-II	5%	5 mL
Hypoxanthine	50 μg/L	2.5 μg
HEPES	25 mM	0.298 g
Glutamax	1X	1 mL
Total	N/A	100 mL

***Note:*** In a microbiological safety cabinet, sterilize the solution using a 0.2 μm filter. Store at 4°C for up to 30 days.

**Table:** 90% Percoll solution

Reagent	Final concentration	Amount
10X RPMI	1X	20 mL
Percoll	90%	180 mL
Total	N/A	200 mL

***Note:*** Dissolve 10.4 g of RPMI-1640 Medium (powder) in 100 mL of sterile distilled water to make 10X RPMI solution. Sterilize the solution using a 0.2 μm filter. Store at 4°C for up to 30 days.

**Table:** 70% Percoll solution

Reagent	Final concentration	Amount
90% Percoll solution	1X	195 mL
13.3% sorbitol-PBS	50 μg/L	55 mL
Total	N/A	250 mL

***Note:*** Store at 4°C for up to 30 days.

***Note:*** Dissolve 7.99 g of sorbitol in 60 mL of 1X PBS to make 13.3% sorbitol-PBS solution. Sterilize the solution using a 0.2 μm filter.

**Table:** TOM-5% KSR

Reagent	Final concentration	Amount
TOM	N/A	47.5 mL
KnockOut Serum Replacement (KSR)	5%	2.5 mL
Total	N/A	50 mL

***Note:*** Store at 4°C for up to 3 days.

**Table:** (0.20%) Trypsin-EDTA

Reagent	Final concentration	Amount
Trypsin (1:250)	0.2% w/v	1000 mg
EDTA	0.2 mg/mL	100 mg
PBS	1X	500 mL

***Note:*** Dissolve in a shaker for 1 h. In a microbiological safety cabinet, sterilize the solution using a 0.2 μm filter. Aliquot 100 mL in sterile glass bottles. Store at −20°C for up to 6 months.

**Table:** Collagenase V stock solution

Reagent	Final concentration	Amount
Collagenase V - 100 mg	10 mg/mL	100 mg
FBS (heat inactivated)	10%	1 mL
PBS	1X	9 mL
Total	N/A	10 mL

***Note:*** Distribute the solution in 1.25 mL aliquots. Store at −20°C for up to 6 months.

**Table:** DNase I stock solution

Reagent	Final concentration	Amount
DNase I	10 mg/mL	100 mg
Sterile water	N/A	10 mL

***Note:*** Distribute the solution in 100 μL aliquots at −20°C for up to 6 months.

**Table:** Collagenase V mix

Reagent	Final concentration	Amount
Collagenase V stock solution	1 mg/mL	2.5 mL
DNase I stock solution	40 μg/mL	100 μL
PBS	1X	22.4 mL
Total	N/A	25 mL

***Note:*** 12.5 mL of this mix is needed to digest one sample. Use fresh.

**Table:** PBS-DNase I

Reagent	Final concentration	Amount
DNase I stock solution	20 μg/mL	100 μL
PBS	1X	49.9 mL
Total	N/A	50 mL

***Note:*** Keep this solution at 4°C or in ice. 45 mL of PBS-DNase I are needed per explant. Use fresh.

**Table:** EDTA 0.5 M solution

Reagent	Final concentration	Amount
EDTA	0.5 M	1.861 g
Distilled water	N/A	10 mL

***Note:*** Gradually add 1.861 g of EDTA to 8 mL of distilled water while stirring. If EDTA does not dissolve completely, adjust pH to 8 by adding a few drops of 1 M NaOH. Use distilled water to adjust the final volume to 10 mL. Store at 4°C for up to 6 months.

**Table:** MACS buffer

Reagent	Final concentration	Amount
MACS BSA Stock Solution	1:20	1 mL
PBS	1X	18.92 mL
EDTA 0.5 M solution	2 mM	80 μL
Total	N/A	20 mL

***Note:*** Keep this solution at 4°C or in ice. 20 mL of MACS buffer are needed per sample. Use fresh.

## Step-by-step method details

**Timing: variable. Infections of placental explants with *L. monocytogenes* require approximately 23 h, *T. gondii* infections need 19 days, and *P. falciparum* infections require 10 days**

***Note:*** The time required for this method varies depending on the pathogen used for the infection.

### Pathogen culture for infection assays

**Timing: variable**

**Timing: 18 h (for step 1)**

**Timing: 8 days (for step 2)**

**Timing: 7–9 days (for step 3)**

This section includes the steps to culture pathogens used to study the placental responses to infections: *L. monocytogenes, P. falciparum*, and *T. gondii*. Make sure you follow the right biosafety measures per each pathogen.

1. Culture of *L. monocytogenes*.
  ***Note:****L. monocytogenes* culture should be performed according to the relevant local biosafety procedures (e.g., containment level 2 in the United Kingdom, biosafety level 2 in the United States of America).
  ***Note:*** The culture of *L. monocytogenes* should start one day prior to infecting placental explants.
  **CRITICAL:** In this protocol we use *L. monocytogenes* EGD-cGFP^2^ a highly pathogenic wild type EGD strain that is constitutively labeled with the fluorescent protein cGFP through chromosomal integration.
  a. Inoculate 10 mL of BHI broth with *L. monocytogenes* EGD-cGFP strain^2^ (Figure 1A).
    i. Culture overnight for 12 h in slanting position at 37°C at 120 revolutions per minute (rpm).

**Figure 1:**
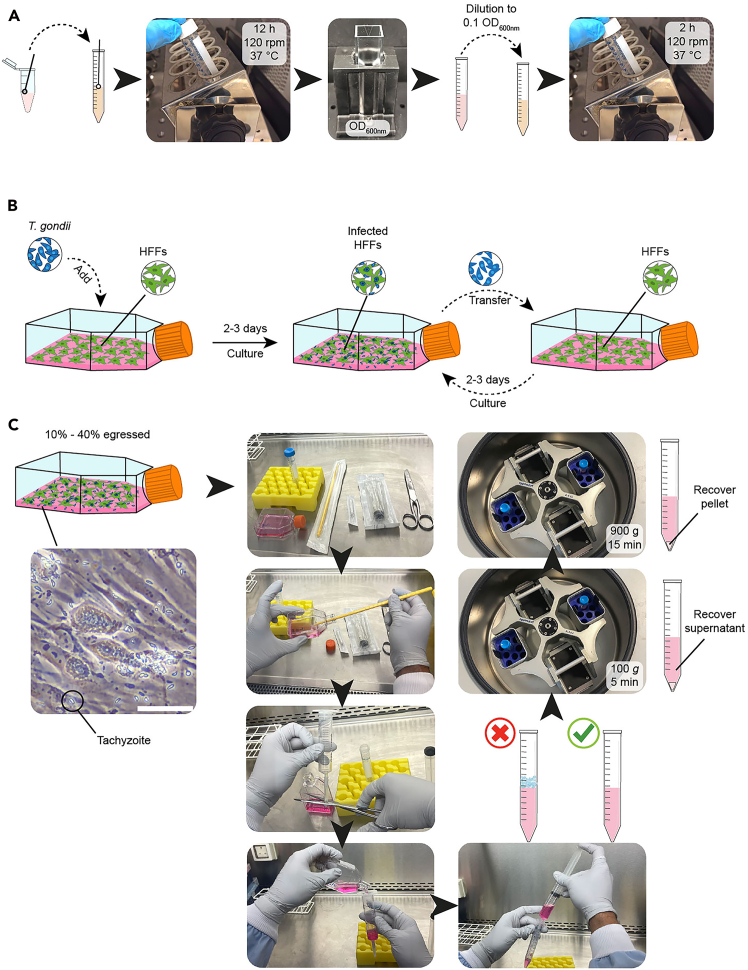
Culture of *L. monocytogenes* and *T. gondii* (A) Culture workflow of *L. monocytogenes* for explant infection. (B) Culture and maintenance of *T. gondii* tachyzoites. (C) Workflow for isolation of *T. gondii* tachyzoites for explant infection. Scale bar: 50 μm.
  b. Measure the optical density at 600 nm (OD_600nm_) after overnight culture, using a spectrophotometer (Biowave CO8000).
    i. Use 1 mL of fresh BHI broth as blank.
    ii. Dilute 100 μL of the overnight culture with 900 μL of fresh BHI broth.
    iii. Take measurement of the diluted overnight culture.
    iv. Multiply the absorbance measurement times the dilution factor (10 in this case), to obtain the OD_600nm_.
  c. Dilute the culture to OD_600nm_ 0.1 using fresh BHI broth.
  d. Incubate the bacteria culture at OD_600nm_ 0.1 for 2 h to ensure reaching the mid-exponential growth phase prior to placental explant infection. This suspension can be used for infection or plated for continuous growing.
2. Culture of *T. gondii* tachyzoites.
  ***Note:****T. gondii* culture should be performed according to the relevant local biosafety procedures (e.g., containment level 2 in the United Kingdom, biosafety level 2 in the United States of America).
  **CRITICAL:** In this protocol we use the *T. gondii* strain RH, which corresponds to a highly virulent wild type line widely used in laboratory research.^4^
  a. Maintain human foreskin fibroblasts (HFFs) in D10 media.
    i. Culture HFFs in T-25 flasks with 5 mL of D10 media in incubator at 37°C (21% O_2_, 5% CO_2_).
  b. Expansion of HFF host cells.
    i. From a pre-existing T-25 of confluent HFFs, aspirate all of the media.
    ii. Add 3 mL of Trypsin-EDTA 0.25% and swirl around to spread the Trypsin-EDTA over all the HFFs.
    iii. Incubate for 60–90 s at 37°C.
    iv. Remove from the incubator and firmly tap the flask several times with a gloved hand to promote HFF detachment from the flask.
      ***Note:*** Hold the flask up to the light to check for successful detachment. Detached HFFs will appear as cloudy white particles floating in the media.
    v. Spray the flask with ethanol and return to the cabinet.
    vi. Quench the Trypsin-EDTA with 7 mL of D10.
    vii. Transfer 500 μL of detached HFFs into new T-25 flasks.
    viii. Add 5.5 mL of D10 media.
    ix. Incubate for 4–5 days at 37°C (21% O_2_, 5% CO_2_) to reach confluency.
  c. Infect confluent HFFs with *T. gondii* in ED1 media (Figure 1B).
    i. Aspirate the media of the yet to be infected HFFs.
    ii. Add 6 mL of ED1 media.
    iii. Transfer 300 μL of parasite-containing media from a flask exhibiting early HFF lysis to fresh HFFs for infection.
      ***Note:*** We recommend utilizing tachyzoites at early stages of host cell lysis—approximately 40–48 h post infection—to minimize prolonged extracellular periods that could negatively affect parasite health.
    iv. Grow parasites for 2–3 days at 37°C (21% O_2_, 5% CO_2_).
    v. Check cultures to see if *T. gondii* has lysed out and pass them as before.
      ***Note:*** Passing the parasites every 2–3 days will maintain their level of effectiveness while keeping them healthy. There is no need to count the tachyzoites in the inoculum.
      ***Note:****T. gondii* tachyzoites duplicate every ∼6 h, bursting out of the host cells in ∼48 h.
      ***Note:*** The passaging timings are based on the RH (wild type) strain and may vary with other strains.
  d. Harvest of *T. gondii* for experimental use using syringe lysis (Figure 1C).
    i. Examine the parasite culture under the microscope and ensure that only 10%–40% of the HFFs have been lysed by the parasite.
      ***Note:*** HFFs should have been infected approximately ∼40 h before harvesting to ensure they have formed large parasitophorous vacuoles containing many parasites, but are yet to lyse the HFF cells.
    ii. Detach infected HFFs using the cell scraper.
    iii. Remove the plunger from a 10 mL syringe (keep sterile).
    iv. Assemble the syringe and 27 gauge needle and prepare a 15 mL tube.
    v. Cut the needle cap such that the needle can eject the liquid without the tip protruding from the cap end.
    vi. Hold the syringe with the needle sitting in the 15 mL tube and decant the detached infected HFFs into the 10 mL syringe.
    vii. Insert the plunger and press slowly until the content is transferred into the 15 mL tube.
      ***Note:*** The shear force created will break the infected HFFs and large parasitophorous vacuoles, releasing the intracellular tachyzoites.
      ***Note:*** Ensure that the liquid flows along the inner wall of the tube to prevent foam formation. If the needle becomes clogged, replace it with a new one.
    viii. Centrifuge the suspension at 100 *g* for 5 min.
      ***Note:*** This step precipitates host cells, but not the parasites.
    ix. Recover the supernatant and centrifuge it at 900 *g* for 15 min.
      ***Note:*** This step pellets the parasites.
    x. Remove the supernatant and resuspend the pellet in 1 mL of Advanced DMEM/F12.
      ***Optional:*** To get extra pure tachyzoites, pass the cell suspension through a 3 μm filter to eliminate host debris. This method yields higher purity at the expense of lower tachyzoite recovery and minimizes the impact of carryover debris on target cells.
  e. Quantify the number of tachyzoites harvested using a Hemocytometer (Neubauer chamber).
    i. Pipette 10 μL of the tachyzoite solution into the hemocytometer.
    ii. Use the 20x lens to count the number of parasites in 5 random squares of the 25 grids with 16 base unit squares at the center of the hemocytometer.
      ***Note:*** The 5 squares must contain at least 100 parasites. If they don't, then count 6 squares instead.
    iii. Obtain the concentration of tachyzoites:

$$Tachyzoites\;per\;mL=Counted\;tachyzoites\times \left(\frac{25\;squares}{squares\;counted}\right)\times 10,000$$

**Figure 2:**
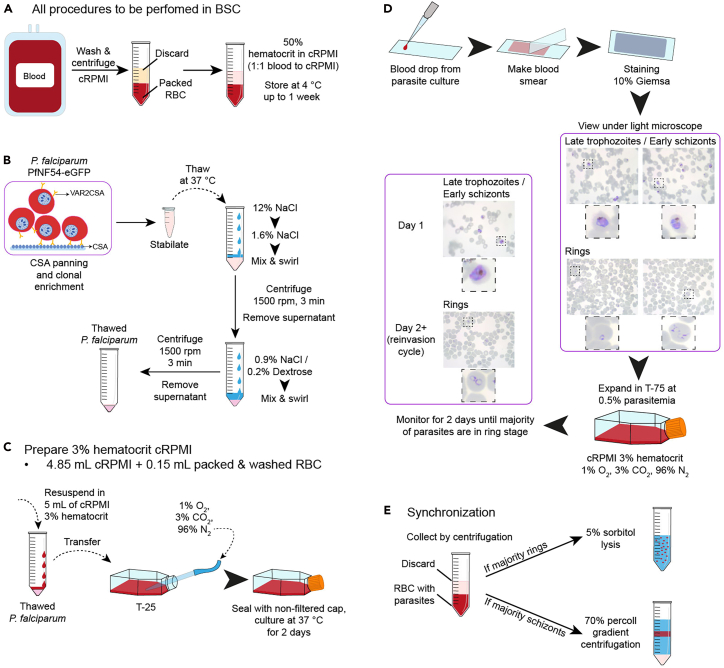
Culture of *P. falciparum* (A) Blood preparation for *P. falciparum* culture. (B) Workflow for thawing frozen *P. falciparum* samples. (C) Initiation of *P. falciparum* culture. (D) Expansion of *P. falciparum* culture. Dashed squares indicate regions shown at higher magnification in the adjacent panels. (E) Synchronization strategies to enrich for ring stage *P. falciparum.* CSA, chondroitin sulfate A; RBC, red blood cells.
  b. Thawing of *P. falciparum* frozen stabilates (Figure 2B).
    i. Allow frozen cryovial containing *P. falciparum* parasites to thaw in a 37°C water or beads bath. Alternatively, thaw frozen cryovial in the 37°C incubator.
    ii. Transfer thawed *P. falciparum* parasites into a 50 mL tube.
    iii. Add 3 drops (approximately 0.2 mL per drop) of 12% NaCl dropwise, swirl and sit for 5 min at RT.
    iv. Add 8 mL of 1.6% NaCl dropwise swirl and mix well and sit at RT for 5 min.
    v. Centrifuge at 1500 rpm for 3 min.
    vi. Remove supernatant but leave a bit of solution.
    vii. Add 8 mL of 0.9% NaCl/0.2% Dextrose dropwise swirl and mix well.
    viii. Centrifuge at 1500 rpm for 3 min.
    ix. Remove supernatant but leave a remnant of solution.
  c. Culture initiation (Figure 2C).
    i. Resuspend thawed *P. falciparum* parasites very gently in 5 mL of cRPMI at 3% hematocrit (0.15 mL of packed RBC) and transfer into a T-25 flask.
    ii. Insert a sterile 1 mL serological pipette to a gas tube and pump gas mixture (1% O_2_, 3% CO_2_ and 96% N_2_) into the T-25 flask for 30 seconds.
    iii. Immediately seal the T-25 flask with a non-filtered cap and culture at 37°C incubator.
    iv. Culture for 2 days.
  d. Culture expansion (Figure 2D).
    i. Take a drop of blood from the parasite culture flask using a p-10 tip, and make a thin blood smear of a clean microscope glass slide. Allow the slide to dry and fix with 100% methanol for 30 seconds. Stain slide with 10% Giemsa for 5 min. Wash off Giemsa with tap water and allow the slide to dry.
    ii. Mount Giemsa-stained slide on a light microscope and count the average of parasitemia in at least 5 different fields of view. Estimate the proportion of different developing life-cycle stages from ring, trophozoite and schizont.
      $$Parasitemia\;\left(\%\right)=\frac{Number\;of\;infected\;RBC}{Total\;number\;of\;RBC}\times 100\%$$
    iii. Expand and dilute parasite culture down 0.5% parasitemia in a T-75 flask with fresh 20 mL cRPMI at 3% hematocrit. Dilute parasitemia by adjusting to the desired final percentage and volume of culture needed, using the washed packed RBC as diluent.
    iv. Insert a sterile 1 mL serological pipette to a gas tube and pump gas mixture (1% O_2_, 3% CO_2_ and 96% N_2_) into the T-75 flask for 30 seconds.
    v. Monitor parasitemia status after 2 days, where the majority of the stages will be rings after one reinvasion cycle.
      **CRITICAL:** Parasites must be synchronized in similar life-cycle stages prior to performing downstream assays. If parasite stages are predominantly rings, proceed to 5-e. Likewise, if parasite stages are predominantly schizont, proceed to 5-f.
  e. Culture synchronization with 5% sorbitol (Figure 2E).
    i. If parasitemia corresponds to ∼5% ring stage, proceed with sorbitol synchronization with at least 20 mL culture (0.6 mL of packed RBC, 3% hematocrit).
    ii. Follow the sorbitol synchronization protocol as detailed in Chapter 1 of Ménard 2012.^5^
    iii. Examine parasitemia status after 2 days.
  f. Culture synchronization with 70% Percoll (Figure 2E).
    i. Prepare 70% Percoll solution for gradient centrifugation.
    ii. Follow the synchronization protocol using gradient centrifugation as detailed in Chapter 2 of Ménard 2012.^5^
    iii. Set up culture expansion as described in 5-d.
    iv. Examine parasitemia status after 18 h where the majority of the parasites will be in the ring stage.

3. Culture of *P. falciparum.*
  ***Note:****P. falciparum* culture should be performed according to the relevant local biosafety procedures (e.g., containment level 3 in the United Kingdom, biosafety level 2/3 in the United States of America).
  **CRITICAL:** In this protocol we use the *P. falciparum* line 2D9 NF54-eGFP, which has been panned repeatedly against chondroitin sulfate A (CSA) to enrich for parasites expressing the receptor *VAR2CSA* that encodes a *P. falciparum* erythrocyte membrane protein 1 (PfEMP1). The high expression of this receptor ensures that *P. falciparum*-infected erythrocytes can properly attach to the human placenta.
  a. Culture media and human blood preparation (Figure 2A).
    i. Make complete RPMI (cRPMI) media.
    ii. Wash human blood 3 times in RPMI 1640 to remove serum and citrate phosphate dextrose. Centrifuge blood in 50 mL tubes at 2,500 rpm to separate serum from red blood cells (RBC) to retain packed RBCs. After the third wash, resuspend the washed packed RBC at 50% hematocrit in cRPMI, where equal volumes of packed RBCs are mixed with cRPMI (1:1 ratio). This can be stored at 4°C for up to 1 week.

### Placental explant setup

**Timing: 60 min**

This section details the setup of the air-liquid interface culture system and the processing of fresh placental tissue to create *ex vivo* explant cultures.

***Note:*** Tissue samples are collected in Ham’s F12 medium and stored on ice immediately after collection. In this protocol we used samples from 5–14 post-conceptional weeks (PCW). For optimal results, the samples should be processed within eight hours of collection.

4. Air-liquid interface culture system setup (Figure 3A).
  a. Thaw 50 μL of Matrigel at 4°C (on ice) per well (ThinCert).
  b. Mix Matrigel with an equal volume of cold TOM (1:1). Use 50 μL of Matrigel and 50 μL of TOM per well (ThinCert).
    i. Keep on ice.
  c. Use a 6 well plate and place the ThinCert transwells into it.
  d. Add 100 μL of Matrigel-TOM mix and create a monolayer on the transwell by spreading the matrigel mix with a pipette tip. Work quickly to avoid premature polymerization. Repeat for each well.
  e. Allow the Matrigel monolayer in the transwells to solidify by incubating at 37°C for ∼15 min.

**Figure 3:**
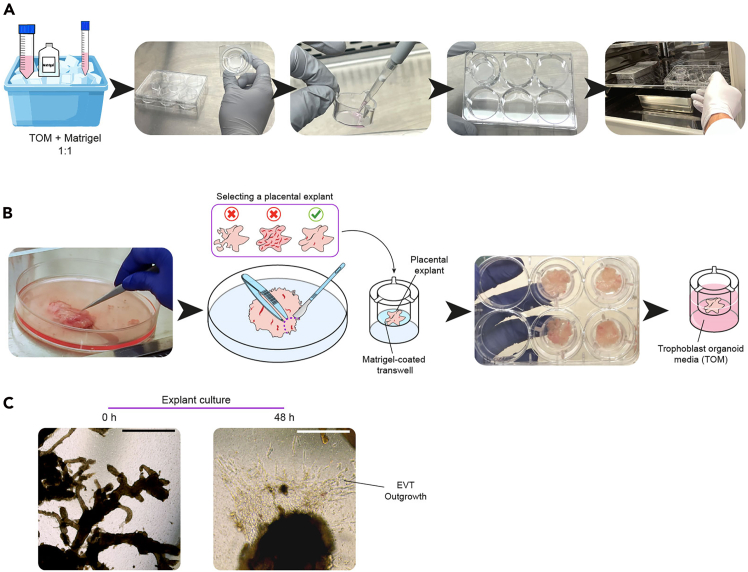
Preparation of placental explants (A) Setup of air-liquid interface culture system. (B) Processing placental tissue to establish placental explants. (C) Placental explants after 0 h and 48 h in culture. Scale bars: 500 μm.
5. Processing of placental tissue (Figure 3B).
  a. Place the collected placental tissue in a sterile petri dish (150 mm).
    i. Aspirate the media.
  b. Wash the tissue twice with 50 mL of ice-cold Ham’s F12 with penicillin-streptomycin.
  c. Remove blood clots in the tissue using the scalpel and forceps.
  d. Select regions for explants by prioritizing areas with abundant villi, minimal blood clots, minimal membranous tissue, and good structural integrity.
  e. Cut the villi in pieces of approximately 1 cm^2^ using the scalpel and forceps.
    ***Note:*** For a clean cut, apply downward pressure with the scalpel on the tissue rather than sliding along it.
  f. Place the tissue in the transwell with the solidified matrigel monolayer.
    i. Let it attach to the Matrigel for 15 min at 37°C.
  g. Add 1.5 mL of TOM in each bottom well (not in the transwells).
    i. Gently add 100 μL of TOM on top of the tissue.
  h. Incubate at 37°C for 12 h.
    **CRITICAL:** Cell viability reduces after 72 h in culture.^1^
    **CRITICAL:** To maintain explants in long-term culture, replace TOM every 2–3 days. A change in media color from pink to yellow indicates the need to replace media.
    ***Note:*** The Matrigel monolayer aids the differentiation of trophoblast in the placental tissue into extravillous trophoblast (EVT),^1^ which invades the Matrigel and forms an outgrowth visible after 48 h (Figure 3C).
    ***Optional:*** To further examine the placental explants, immunofluorescence assays can be performed using antibodies against specific markers such as CD138 (Syndecan-1, syncytia marker), HLA-G (EVT marker), CD64 (Hofbauer cell marker), CD45 (immune cell marker), or Vimentin (fibroblast marker).^1^

### Infection of placental explants

**Timing: 90 min**

This section includes the steps to infect placental explants using *L. monocytogenes*, *T. gondii* and *P. falciparum*.

6. Infection with *L. monocytogenes*.
  ***Note:*** TOM-Lm is required to perform placental infections with *L. monocytogenes*. Unlike the original TOM, TOM-Lm contains Amphotericin B instead of Primocin to prevent fungal infections without killing the bacteria.
  a. Warm PBS supplemented with 4 μg/mL of Amphotericin B to 37°C.
  b. Prepare the antibiotic free TOM-Lm warm to 37°C.
  c. Prepare the *L. monocytogenes* culture at exponential growth phase.
  d. Aspirate media from the bottom transwell.
  e. Rinse inner (ThinCert) and bottom transwells twice by gently pipetting 1 ml of warm supplemented PBS, carefully aspirate the PBS.
  f. Gently pipette 1 mL TOM-Lm to inner and bottom wells. Be careful not to disturb the explants.
  g. Incubate the explants in TOM-Lm for 1 h to ensure that antibiotics have been removed.
  h. Prepare *L. monocytogenes* inoculum at 1.5x10^8^ colony forming unit (CFU)/mL in TOM-Lm for infection with 1.5x10^8^ CFU of bacteria per explant. 1 mL of inoculum is needed per explant.
    i. Measure the OD_600nm_ as described in step 1-b.
    ii. Adjust the concentration to 1.5x10^8^ CFU/mL in TOM-Lm. OD_600nm_ is equal to 1x10^9^ CFU/mL.
  i. Aspirate media from inner transwells. Gently add 1 mL of inoculum into the inner well.
    i. Keep the same media from the bottom well.
    ii. Incubate plates for 5 h at 37°C (21% O_2_, 5% CO_2_) to allow for bacterial invasion.
  j. Remove extracellular bacteria by aspirating media from inner and bottom wells.
    i. Rinse wells twice with 1 mL of warm supplemented PBS.
  k. Add 1 mL of TOM-Lm supplemented with Gentamicin to the inner well and another 1 mL to the bottom. Incubate plates at 37°C (21% O_2_, 5% CO_2_) overnight.
    ***Note:****L. monocytogenes* is an intracellular bacterium. Gentamicin has a bactericidal effect on extracellular *L. monocytogenes*, allowing only intracellular *L. monocytogenes* to grow and spread through the organ culture.
  l. On the next day, aspirate TOM-Lm containing gentamicin.
  m. Add 1.5 mL of fresh TOM-Lm per well. Incubate plates at 37°C (21% O_2_, 5% CO_2_) for up to 48 h.
  n. Collect supernatant and store it in −80°C for further experiments.
    ***Optional:*** To visualize the infected placental explants, immunofluorescence assays can be performed using antibodies against CD138 (Syndecan-1, syncytia marker), CD64 (Hofbauer cell marker), HLA-G (EVT marker), or CD45 (immune cell marker), and *L. monocytogenes.*^1^
7. Infection with *T. gondii* tachyzoites.
  ***Note:*** TOM-Tg is required to perform placental infections with *T. gondii*. Unlike the original TOM, TOM-Tg contains Amphotericin B instead of Primocin to prevent fungal infections without affecting *T. gondii*. Additionally, TOM-Tg does not include A83-01, as we observed that this component interferes with the parasite's replication inside the host cell.
  a. Warm at 37°C PBS supplemented with 4 μg/mL of Amphotericin B.
  b. Prepare the antibiotic free TOM-Tg, warm to 37°C.
  c. Prepare *T. gondii* inoculum at 1x10^6^ parasites/mL in TOM-Tg. 1 mL of inoculum is needed per explant.
    i. Quantify the tachyzoites as described in step 2-e.
    ii. Adjust the concentration to 1x10^6^ parasites/mL.
  d. Aspirate media from the bottom of the transwell with the explant.
  e. Rinse inner and lower transwells twice by gently pipetting 1 ml of warm supplemented PBS, carefully aspirate the PBS.
  f. Gently pipette 1 mL TOM-Tg to inner and bottom well. Be careful not to disturb the explants.
  g. Aspirate media from inner transwells. Gently add 1 mL of inoculum into the well.
    i. Keep the same media from the bottom well.
    ii. Incubate plates for 5 h at 37°C (21% O_2_, 5% CO_2_) to allow for parasitic invasion.
  h. Remove extracellular parasites by aspirating media from inner wells.
    i. Rinse wells twice with 1 mL of warm supplemented PBS.
      ***Note:****T. gondii* tachyzoites cannot cross from the inner well into the bottom well.
  i. Add 1.5 mL of fresh TOM-Tg to each bottom well. Incubate plates at 37°C (21% O_2_, 5% CO_2_) for up to 48 h.
  j. Collect supernatant and store it in −80°C for further experiments.
    ***Optional:*** To visualize the infected placental explants, immunofluorescence assays can be performed using antibodies against CD138 (Syndecan-1, syncytia marker), CD64 (Hofbauer cell marker), HLA-G (EVT marker), or CD45 (immune cell marker), and *T. gondii.*^1^
8. Infection with *P. falciparum.*
  ***Note:*** TOM-Pf is required to perform placental infections with *P. falciparum*. Unlike the original TOM, TOM-Pf contains 5% hematocrit necessary for infection with *P. falciparum* and it is depleted of the antimicrobial reagent Primocin.
  a. Warm at 37°C TOM-5% KSR.
  b. Warm at 37°C PBS.
  c. Collect synchronized early ring stage (5–10 h rings) cultures at high parasitemia (>4%) by centrifugation at 1500 rpm for 3 min (from 5-e or 5-f). Wash the infected RBC pellet twice with TOM-5% KSR by centrifugation at 1500 for rpm 3 min.
    ***Note:*** We consider the number of packed RBC to be around 10^10^/mL (or 10^7^/μL). Therefore, at 5% parasitemia, there will be approximately 5x10^8^ parasites/mL at 3% hematocrit (or 5x10^5^ parasites/μL of packed RBC). This value is according to the counted parasitemia at step 3-d.
    ***Note:*** We infect explants with 3x10^5^ parasites per μL of RBC, selected according to the World Health Organization (WHO) definition of hyperparasitemia (>2.5x10^5^ parasites/μL of RBC) in areas of high malaria transmission.^6^
  d. Dilute the final concentration of infected RBC to 3x10^8^ parasites/mL (or 3x10^5^ parasites/μL of packed RBC) with washed RBC to a final blood volume of 0.03 mL per explant to infect.
  e. Use the infected RBC to prepare 1 mL TOM-Pf per explant to infect.
    ***Note:*** Scale up the volumes accordingly for the desired number of infected wells.
  f. Rinse inner and lower trans wells three times by gently pipetting 1 mL of warm supplemented PBS, carefully aspirate the PBS.
  g. Gently pipette 1 mL TOM-Pf to the inner well, as *P. falciparum* is non-motile at blood stage. Add 1 mL of TOM-Pf to the bottom well. Be careful not to disturb the explants.
  h. Incubate plates for 18–20 h at 37°C (21% O_2_, 5% CO_2_) for *P. falciparum* to progress to trophozoite stage.
  i. Collect supernatant and store it in −80°C for further experiments.
    ***Optional:*** To visualize the infected placental explants, immunofluorescence assays can be performed using antibodies against CD138 (Syndecan-1, syncytia marker), CD64 (Hofbauer cell marker), HLA-G (EVT marker), or CD45 (immune cell marker), and Green fluorescence protein (GFP; for our *P. falciparum* 2D9 NF54-eGFP strain containing GFP).^1^

### Dissociation of placental tissue into single-cell suspension

**Timing: 90 min**

This section describes how to dissociate the placental tissue either from fresh placenta or explants into a single cell suspension that can be used to run single-cell genomics techniques.

9. Processing placental tissue.
  a. Placental Explants (Figure 4A):
    i. Gently wash the explant in the transwell with 1 mL of ice-cold PBS. Remove the supernatant.
    ii. Repeat the washing step above.
    iii. Incubate the explant with 1 mL of ice-cold cell recovery solution for 15 min on ice, shake the plate every 5 min. Explants should dislodge from the transwell.
    iv. Transfer the explant to a small petri dish (60 mm).
    v. Transfer to the small petri dish with a pipette the remaining cell recovery solution in the transwell.
    vi. Rinse the transwell with 1 mL of ice-cold PBS. Then, collect and transfer the rinsing solution into the small petri dish.
      ***Note:*** Steps 9-a-v and 9-a-vi help to maximize the recovery of EVT cells.
    vii. Hold down one end of the placental tissue and gently scrape the villi from the chorionic membrane with a scalpel (Figure 4A).

**Figure 4:**
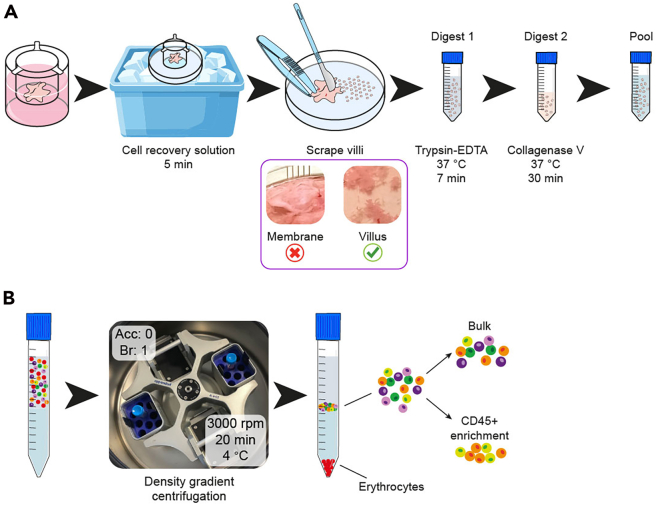
Dissociation of placental explants into single-cell suspension (A) Placental dissociation workflow to obtain single-cell suspension. (B) Density gradient centrifugation workflow to remove erythrocytes from the cell suspension using Pancoll. Acc, acceleration; Br, brake.
  b. Fresh placental tissue (optional).
    ***Note:*** This step is only necessary if you need to process fresh tissue for control experiments, not explant tissue.
    i. Process the placenta immediately upon receiving.
    ii. Rinse the placenta in 1X Ham’s F12 gently with a magnetic stirrer for 10 min at room temperature (RT).
    iii. Transfer the placenta to a petri dish (60 mm).
    iv. Remove any blood clots with forceps.
    v. Hold down one end of the placental tissue and gently scrape the villi from the chorionic membrane with a scalpel.
10. Digestion and cell suspension preparation (Figure 4A).
  a. Transfer scraped villi with a Pasteur pipette (with the tip cut off) in 40 mL of (0.20%) Trypsin-EDTA pre-warmed at 37°C in a 50 mL Falcon tube sealed with parafilm.
  b. Place the solution in an incubator at 37°C and rotate gently for 7 min (digest 1).
  c. Add 1 mL FBS per 40 mL of digestion mix to stop the trypsinization.
  d. Filter the trypsinized tissue through a 70 μm strainer, rinsing with 5–10 mL of PBS-DNase I at RT. Save the filter with the undigested tissue.
  e. Centrifuge the filtrate for 5 min at 200 *g* to pellet the cells. Discard the supernatant and keep the pellet on ice.
  f. Transfer undigested tissue from the filter using forceps to a 50 mL Falcon tube.
  g. Prepare a Collagenase V mix for digestion. Use 12.5 mL of Collagenase V mix per 1 cm^2^ explant.
    ***Note:*** The addition of DNase I in the Collagenase V mix is crucial for macrophage viability.
  h. Digest each explant in 12.5 mL of Collagenase V mix for 30 min at 37°C on a heated shaker at 500 rpm (digest 2).
  i. Filter the resultant cell suspension through a 100 μm strainer and wash through with additional 5 mL of PBS.
    i. Discard undigested tissue.
  j. Centrifuge the filtrate for 5 min at 200 *g* at RT, to pellet the cells.
    i. Discard the supernatant and keep the pellet in ice.
  k. Pool the cells from digest 1 and digest 2, wash with 5 mL of PBS-DNase I, and centrifuge for 5 min at 200 *g* at RT.
    ***Note:*** Pooling these fractions maximizes the yield of Hofbauer cells, as maternal and trophoblast cells are primarily present in the initial digest.
  l. Resuspend the cell pellet gently in 1 mL PBS-DNase I, pass through a 40 μm strainer. Keep the cell suspension on ice.
  m. Count cells with a hemocytometer using 5 μL trypan blue and 5 μL sample.
    ***Optional:*** Cell viability can also be assessed using a LIVE/DEAD Fixable Near-IR Dead Cell Stain in conjunction with fluorescence-activated cell sorting (FACS).
11. Pancoll density gradient separation for erythrocyte removal (Figure 4B).
  a. Resuspend the cell pellet in 10 mL of PBS-DNase I.
    ***Optional:*** 20 mL for big fresh placental samples.
  b. Gently layer the cell suspension onto 5 mL of Pancoll in a 15 mL Falcon tube by tilting the tube 45° and adding the cell suspension with a Pasteur pipette.
    ***Optional:*** For large fresh placental samples use 10 mL of Pancoll in a 50 mL Falcon tube.
    **CRITICAL:** Ensure that the Pancoll and cell suspension do not mix. Two layers must be formed.
  c. Centrifuge for 20 min at 3000 rpm (or 600 *g*), 4°C, with acceleration 0 and brake 1.
    **CRITICAL:** The right acceleration and braking settings are fundamental to successful gradient separation. Temperature must be 4°C to maximize cell viability.
  d. Collect cells at the interface with a Pasteur pipette and transfer to a 15 mL tube.
    ***Note:*** Erythrocytes precipitate while other cell types form a white layer at the Pancoll-suspension interface.
  e. Wash the collected cells with 10–15 mL of PBS-DNase I.
    i. Centrifuge for 5 min at 200 *g*, RT.
    ii. Check the pellet and discard the supernatant.
  f. Repeat the washing step.
  g. Resuspend cells in 1 mL of PBS-DNase I and pass through a 40 μm strainer. Keep the single-cell suspension in ice.
    ***Note:*** If there is excessive cell debris in the solution, pass it through a 20 μm strainer to improve the quality of the sample.

### Immunomagnetic CD45+ enrichment

**Timing: 50 min**

This section outlines the process for enriching CD45+ immune cells from the single-cell suspension using immunomagnetic isolation. This enrichment step is particularly useful for studying Hofbauer cells, the only immune cell population present within the placental stroma. Since Hofbauer cells are less abundant than other cell types, such as fibroblasts and trophoblasts, this enrichment step enables a higher recovery of Hofbauer cells. A portion of the previously prepared single-cell suspension is allocated specifically for this purpose, while the remaining suspension can be reserved for analyzing other cell types of interest.

12. Cell magnetic labeling for immunomagnetic enrichment.
  a. Separate half of the cells recovered after the Pancoll separation for enrichment, ideally 500,000 cells. Keep cells on ice.
    i. Keep remaining cells in ice.
  b. Prepare MACS buffer and keep ice-cold.
  c. Centrifuge cells suspension for 10 min at 300 *g.*
    i. Remove all the supernatant without disturbing the pellet.
  d. Resuspend cell pellet in 80 μL of MACS buffer.
  e. Add 20 μL of CD45 MicroBeads.
    i. Use a micropipette to mix well.
    ii. Incubate 15 min in ice or at 4°C.
      **CRITICAL:** To ensure a successful separation, keep cells cold and pre-cool the MACS buffer.
  f. Wash the cells by adding 1 mL of MACS buffer.
    i. Centrifuge for 10 min at 300 *g.*
    ii. Resuspend cells in 500 μL of MACS buffer.
13. Magnetic separation with LS columns.
  a. Place LS columns in the magnetic QuadroMACS separator.
    ***Note:*** One LS column is required per sample.
  b. Prepare the column by rinsing it with 3 mL of MACS buffer.
  c. Transfer the suspension of magnetically labeled cells into the column.
  d. Collect the unlabeled fraction that has passed through the column.
  e. Wash the column with 3 mL of MACS buffer.
    i. Repeat the washing step 2 more times.
  f. Remove the column from the QuadroMACS separator.
    i. Place the columns in a collection tube of at least 5 mL.
  g. Add 5 mL of MACS buffer into the column.
    i. Immediately flush out the cells by firmly pushing the plunger into the column.
    ii. Keep cells in ice.
      ***Note:*** This fraction corresponds to the magnetically labeled cells.
  h. Centrifuge the magnetically labeled cells for 5 min at 200 *g*, RT.
  i. Gently resuspend the pellet in 100 μL of PBS-DNase I. Keep cells in ice.
  j. Count cells with a hemocytometer using 5 μL trypan blue and 5 μL sample.
    ***Note:*** If there is excessive cell debris in the solution, pass it through a 20 μm strainer to improve the quality of the sample.

## Expected outcomes

This protocol provides a comprehensive workflow for modeling infections caused by *T. gondii*, *P. falciparum*, and *L. monocytogenes* using placental explants. The expected outcomes include culturing all three pathogens (Figures 1 and 2), establishing multilineage placental explants (Figure 3), conducting infection assays with *L. monocytogenes*, *P. falciparum*, and *T. gondii*, dissociating the tissue to produce a single-cell suspension (Figure 4A), and enriching for CD45+ cells to preserve the immune cell compartment in the final sample (Figure 4B).

The resulting infected and uninfected explants are versatile and can be used for downstream applications. These include freezing in optimal cutting temperature (OCT) compound for cryosectioning and imaging, flash freezing for single-nuclei isolation, or preparing single-cell suspensions for single-cell transcriptomics using platforms such as 10x Genomics or for FACS analysis.

## Limitations

This protocol is designed to maintain stable cell viability of the tissue for up to 72 h. As such, the explant system is not suitable for longitudinal infection monitoring or studying chronic infections. To extend tissue viability for long-term cultures, placental explants can be integrated with microfluidic chambers or organ-on-chip systems. Moreover, under the current explant culture conditions, *P. falciparum* cannot progress to the next life cycle of reinvading RBCs. Further method improvement is required to evaluate the effect of long-term malaria infection.

## Troubleshooting

### Problem 1

Insufficient intracellular *T. gondii* infection.

### Potential solution

Make sure that TOM-Tg prepared in 7-b has been used during the infection steps. The reagent A83-01, used in regular TOM, TOM-Lm and TOM-Pf, affects *T. gondii* replication.

### Problem 2

After performing section 9 and 10, incomplete tissue dissociation was observed.

### Potential solution

Ensure the tissue is adequately minced during 9-a-vii or 9-b-v, this is vital to increase cell recovery. Additionally, in 10-a and 10-h incubate with the indicated enzyme concentrations for the full digestion time. Small fragments of non-dissociated tissue after the Collagen V digestion in 10-i are expected.

### Problem 3

Low cell viability is observed following cell suspension preparation in steps 10 and 11.

### Potential solution

Keep cell suspensions obtained after section 10 on ice at all times, and work quickly to maintain cell viability. Use PBS-DNase I at every indicated step in sections 10, 11 and 12 to minimize macrophage death. Centrifuge cell suspensions at 4°C. Note that some placental samples may exhibit higher levels of apoptosis than others; carefully assess the sample’s condition before beginning the explant setup.

### Problem 4

Pancoll density gradient centrifugation on section 11 does not separate RBCs.

### Potential solution

Ensure the acceleration and brake settings are correctly configured as described in 11-c. Verify that the cells are spun at 3000 rpm for a full 20 min in 11-c. We have noted that some centrifuges include acceleration time in the total spin duration; since acceleration must be slow, this may significantly reduce the actual time the cells are spinning at the target speed. Adjust the timing accordingly to ensure proper centrifugation.

### Problem 5

Recovered cells do not pellet in step 11-e after Pancoll separation.

### Potential solution

If an excess of Pancoll is collected along with the cells during 11-d, it can alter the solution’s density and prevent proper pelleting of the cells. To resolve this, add additional PBS-DNase I during 11-e to dilute the Pancoll as much as possible before centrifugation. This should allow the proper separation of cells.

## Resource availability

### Lead contact

Further information and requests for resources and reagents should be directed to and will be fulfilled by the lead contact, Roser Vento-Tormo (rv4@sanger.ac.uk).

### Technical contact

Technical questions on executing this protocol should be directed to and will be answered by the technical contact, Elias R. Ruiz-Morales (err35@cantab.ac.uk).

### Materials availability

This study did not generate new unique reagents.

### Data and code availability

This study did not generate/analyze datasets or code.

## Acknowledgments

This publication is part of the Human Cell Atlas. We gratefully acknowledge the Cellular Genetics Wet Lab Support Team for their help in the wet lab logistics and Nick Thomson and Sally Kay for bacteria culture facilities. Placental material was provided by the Joint MRC-Human Cell Atlas (MR/S036350/1). We thank the patients for donating tissue for research. This research was funded by the Wellcome Trust grants 206194 and 220540/Z/20/A and the UK Research and Innovation (UKRI) under the UK government’s Horizon Europe funding guarantee (grant number EP/Y009924/1). Additional support to R.F.W. was received from the Wellcome grant 214298/Z/18/Z. We are also thankful to the IBSA Foundation for scientific research for supporting E.R.R.-M. with an IBSA Foundation Fellowship.

## Author contributions

R.V.-T. and R.H. conceived the establishment of placental explants with contributions from E.R.R.-M.; E.R.R.-M. and R.H. set up and characterized the explant system; D.F.-A. provided scientific advice on the use of transwells and air-liquid interface culture for placental explants; *P. falciparum* cultures were performed by R.H. with advice from M.C.S.L.; *T. gondii* culture was performed by E.R.R.-M. following the protocols and guidance of R.F.W.; *L. monocytogenes* cultures were done by R.H. and E.R.R.-M.; I.K. supported the experiments; R.V.-T. supervised the work; E.R.R.-M. wrote the manuscript with contributions from R.H. and R.V.-T.; E.R.R.-M. made the figures. The final version of the manuscript has been approved by all the authors.

## Declaration of interests

The authors declare no competing interests.
